# A multilevel analysis of individual, household and community level factors on stunting among children aged 6–59 months in Eswatini: A secondary analysis of the Eswatini 2010 and 2014 Multiple Indicator Cluster Surveys

**DOI:** 10.1371/journal.pone.0241548

**Published:** 2020-10-30

**Authors:** Maswati S. Simelane, Garikayi B. Chemhaka, Eugene Zwane

**Affiliations:** Department of Statistics and Demography, Faculty of Social Sciences, University of Eswatini, Kwaluseni, Eswatini; Helen Keller International, SIERRA LEONE

## Abstract

**Introduction:**

Child stunting is a significant public health problem in Eswatini. It is associated with a range of child health outcomes, including morbidity, physical and cognitive growth.

**Objective:**

To determine the individual, household, and community-level factors associated with child stunting in Eswatini in 2010 and 2014.

**Methods:**

Using the Eswatini Multiple Indicator Cluster Surveys conducted in 2010 and 2014, a secondary analysis was done of the children surveyed, aged 6–59 months. A total of 1,891 were surveyed in 2010, and 1,963 children in 2014. Univariate, bivariable analysis and multivariable multilevel logistic regression were used to establish the factors associated with stunting.

**Results:**

The study found that stunting decreased significantly between 2010 and 2014, from 31.4% to 25.5% (p<0.001). In both 2010 and 2014, lower odds of stunting were observed among female children, in children born to women with tertiary education compared to those born to women with no formal education. Lower odds of stunting were observed among children from rich households compared to poorest households. In both 2010 and 2014, increased odds of stunting were observed among children aged 12–23, 24–35 and 36–47 months compared to children aged 6–11 months. At the household level, higher odds of stunting were observed among children from households with two and more children under five years of age compared to those with only one child and in 2010, among children from households with a pit latrine and no toilet facility compared to households with a flush toilet. At the community level, in 2010, higher odds of stunting were observed among children from the Shiselweni compared to those from the Lubombo region.

**Conclusion:**

The findings highlight the individual, household, and community-level factors significantly associated with stunting and the changes between the two surveys.

## Introduction

Globally, much has been done to reduce child mortality from about 12.5 million in 1990 to about 5.2 million in 2019 [1]. Regardless of the positive progress made in reducing child mortality globally, differences still exist with countries in Sub-Saharan Africa (SSA) experiencing the highest under five mortality, approximately 2.8 million under five deaths (53% of the global share) in 2019 [1, 2]. In 2019, Nigeria, Congo, Ethiopia, United Republic of Tanzania, and Angola were listed among the top ten countries with the highest child mortality. The persistent under five morbidity and mortality is a result of preventable diseases and clinical conditions, including stunting. Stunted children are five times more likely to die than non-stunted [3, 4].

To prevent the adverse health effects of poor nutrition and multiple recurrent infections with late or inadequate management, the World Health Assembly (WHA) established six pillars of targets for 2025, including stunting [5]. The WHA objective is to reduce stunting by 40% by the year 2025 [5]. In 2015 stunting and other nutrition indicators were incorporated into the global agenda of Sustainable Development Goals (SDGs) to be achieved by the year 2030, goal number two of these SDGs targets ending hunger, achieving food security and improving nutrition, and promoting sustainable agriculture [6].

Developing countries such as Eswatini recorded a reduction of child stunting of 5% from 2010 to 2014, from a prevalence of 30.9% in 2010 to 25.3% in 2014 among children under two years [7, 8]. Previous studies established in developing countries that some of the factors associated with child stunting include: the age of the child; sex of the child; maternal age; maternal education; the number of children under five years in the household; the source of drinking water; the household wealth index; the place of residence; and the region of residence [9–11]. Even though studies investigating factors associated with child stunting have been conducted in other developing countries such as Zambia, Ghana, Tanzania, and Ethiopia [12–15] in Eswatini, only descriptive reports serve as a source of information for stunting [7, 8]. Investigating the factors associated with stunting is beneficial for countries as it provides them with data to assess the impact of national efforts towards meeting the SDG targets. Children are nested within the household, and households are located in the community; therefore, multilevel methods are more appropriate for analysis of hierarchical data [15–17].

### Country context

Eswatini is a landlocked country in Southern Africa surrounded by the Republic of South Africa and Mozambique, measuring 17,364 km^2^, with a population of about 1,093,238 million, 531,111 males and 562,127 females, 78% of whom live in rural areas [18]. The overall growth rate of the population between 2007 and 2017 was 0.7%, not very different from the 0.9% obtained between 1997 and 2007 [19]. Eswatini has experienced a rapid fertility decline over the years, with 3.3 children per woman in 2014 [7, 19]. The country has a per capita Gross Domestic Product (GDP) of US$ 2,776, with the main drivers of the economy being agriculture and manufacturing. Of the total population, 53.5% of children aged 0–14 years, 64.1% out of all those aged 15–24 years, and 54.1% of all those aged 15–35 years live below the poverty line of US$1.90 per day [2, 20]. Overall, in 2017, 58.9% of the population lived below the poverty line, a decrease of 4.1% from 2010. The literacy level stands at 87.5%, while unemployment stands at 28.1%, with about 44% of women and 52% of men benefiting from paid employment. Eswatini experienced a substantial increase in underfive mortality from 67.4 per 1000 live births in 1990 to 116.7 per 1000 live births in 2005, and a sharp reduction was observed to 67 deaths per 1,000 live births in 2014 and 49.4 deaths per 1000 live births in 2019 [7, 21]. Evidence from the 2014 Multiple Indicator Cluster Survey (MICS) report shows that malnutrition in Eswatini is also responsible for the mortality of under-five children along with other infectious diseases [7]. The problem of malnutrition was pervasive in 2009 and costed the country about US$92 million, equivalent to 3.1% of the country’s GDP. To address the problem of malnutrition, in 2017, UNICEF in Eswatini trained 90 health care workers (HCW) from 9 health facilities and 324 community motivators to strengthen institutional and community capacity to deliver malnutrition prevention and treatment services which aimed to facilitate community-based nutrition promotion, screening and early referral of malnourished children to health facilities [22]. Several other initiatives have been put in place by the Eswatini government to combat child morbidity and mortality, they include increasing access to quality ante-natal care services in line with the WHO recommendation [23, 24], safe delivery, immediate and exclusive breast feeding [7], timely and adequate complementary feeding, timely and appropriate management of neonatal, infant and childhood illnesses and poverty alleviation [25], access and improved contraceptive use and keeping young girls in schools [19, 26] and access to improved water and sanitation [22, 27, 28].

Even though one of the goals set out in the SDGs includes achieving good health for all and zero hunger, the country is still far behind in achieving the targeted neonatal mortality rate to at least 12 deaths per 1,000 live births and under-5 mortality to at least as low as 25 deaths per 1,000 live births by the year 2030 [6]. Therefore, by examining the individual, household, and community-level factors on stunting among children, stakeholders may identify the important factors to inform programming. However, there is a scarcity of published scholarly studies from Eswatini that could provide data to inform programming related to child stunting. For that reason, this study was conducted to 1) describe the prevalence of child stunting in 2010 and 2014 in Eswatini, and 2) identify the determinants and random effects of child stunting in Eswatini in these years.

## Materials and data sources

This study was a secondary analysis of data from the 2010 and 2014 Eswatini Multiple Indicator Cluster Surveys (MICSs). The MICS is an international initiative by the United Nations Children's Fund (UNICEF) to assist countries in collecting and analyzing data for monitoring the situation of children, women, and men in developing countries. It is a cross-sectional household survey conducted every three to five years to enable countries to capture rapid changes in key indicators such as those related to health, education, and development. In the Eswatini Multiple Indicator Cluster Survey (EMICS) data was collected using standardized survey questionnaire through face-to-face interviews among nationally representative samples of households. The EMICS was representative and collected information on households, men, women, and children. The sampling frame of the enumeration was based on the 2007 Eswatini Population and Housing Census [21]. A two-staged sampling technique and a systematic sampling technique were applied. First, enumeration areas (EAs), also known as the primary sampling unit (PSU), were selected. Second, households were selected stratified by the rural and urban residence and the four regions of the country, which are Manzini, Hhohho, Shiselweni, and Lubombo [7, 8]. To collect data for children under five years, a standardized questionnaire was used to collect information for each child in the selected households. The mother or caregiver was the respondent for the child questionnaire [7, 8].

### Study design

For the 2010 EMICS, there were 5,475 households selected from 345 enumeration areas (EA). Among the sampled households, a total of 4,834 were successfully interviewed, which included 4,956 women aged 15–49 years and 4,646 men aged 15–59 years. The overall household response rate was 95%.

In the 2014 EMICS, a total of 347 EAs and 5,211 households were selected for the survey. Within each region, the urban and rural areas were identified as the main sampling strata. Within each stratum, a sample of 15 households was selected systematically using probability proportional to size (PPS) in each EA, and a specified number of census EAs were chosen systematically using PPS [7]. A total of 4,762 women (aged 15–49 years) and 1,459 men (aged 15–59 years) were successfully interviewed. A detailed description of the sampling design is available in the 2014 MICS report [7].

In this study for both surveys, 2010 and 2014, mothers and child caregivers were interviewed on the health of children aged 6–59 months to collect data on child stunting. In the 2010 sample, 2,378 children aged 6–59 months had questionnaires completed about them during the interviews. However, of those, only 1,891 had complete information provided and were included as the final sample. In the 2014 EMICS, a total of 2,458 children aged 6–59 months had questionnaires completed about them during the survey interviews. However, 52 children had their height for age z scores not measured and 443 women/caregivers had missing data on their age; hence were excluded from the analysis. Therefore, the final sample included 1,963 children with complete data.

#### Outcome variable

The outcome variable for the study was child stunting, measured in height for age during the survey. The following cut-offs defined by the World Health Organization (WHO) were used: stunted *<* -2 HAZ; moderately stunted ≥-3 HAZ *<* -2; severely stunted *<*-3 HAZ, in line with previous studies [16, 29–31]. A binary variable was created to define stunting: “not stunted” was defined by a Z-score greater than or equal to -2 SD, and “stunted” was defined by a Z-score less than -2 SD. Though the indicator was simplistic, the definition reflected on the assessment of the dynamics of growth and could be used consistently over the whole growth trajectory [32].

#### Explanatory variables

Several factors at the child level, maternal level, household, and community level were found to be associated with stunting [10, 15, 29, 30]. At the child level, the factors were the age of the child and the child’s sex. At the maternal level, the factors were maternal age, maternal education, parity, and marital status. The household factors were the household wealth index, the source of drinking water, the type of toilet facility, the number of children under five years in the household and electricity in the household. The community-level factors were the area of residence, the region of residence, community poverty, and the proportion of women with less than secondary education in the community. The community variables were derived from the individual and household level variables and categorized as low, medium, or high, as done in previous studies [29, 30].

### Statistical analysis

Stata 15 (Stata Corp, Texas, USA) was used for the analysis. First, univariate analysis was used to describe the sample and the weighted prevalence of stunting by survey year. Second, bivariable analysis with Chi-square statistics was performed to test the independence of the distribution between the independent variables and the dependent variable. A two-sample, two-tailed z-test was performed to determine the difference in the weighted proportions of child stunting between the two survey years. The survey weights were applied in all analyses due to the hierarchal nature of the EMICS survey. Third, a bivariable and multivariable multilevel logistic regression was used to identify the factors that were significantly associated with child stunting. For the multilevel analysis, a two-level model was used to report the random (measures of variation) and fixed effects (measures of association) estimates of the model. Five models were specified for the study: model 1 (empty model) included only the outcome variable, model 2 included only the individual factors; model 3 included the household level factors; model 4 included only the community-level factors; and model 5 included the individual, household and community-level factors in one model. The fixed effects results were reported as adjusted odds ratios (AOR) at a 95% confidence interval (95% CI). The intraclass correlation coefficient (ICC) [33], and the proportion change in variance (PVC) were used to report the variation of stunting at the community level. The Akaike information criterion (AIC) was used to test the models.

### Ethical consideration

The UNICEF team granted permission for the access and use of the EMICS dataset from http://mics.unicef.org/surveys. They are anonymous and do not allow the identification of participants. The data is publicly available and the authors had no special access privileges to the data and that other researchers will be able to access the data in the same manner as the authors.

## Results

A total of 1,891 children were included in the analysis in 2010 and 1,963 in 2014. There was a similar distribution of the child-level factors: age group and sex, maternal-level factors: age group, marital status, maternal education and parity, household factors: household wealth and community-level factors: region of residence, community poverty, and the proportion of mothers with lower than secondary education level as shown in Table 1.

**Table 1:** Sample characteristics and distribution of child stunting by the explanatory variables, 2010 and 2014.

	2010 (n = 1,891)	2010 Stunted (n = 593)		2014 (n = 1,963)	2014 Stunted (n = 512)	
**Variables**	n (%)	n (%)	P-value (Chi-square)	n (%)	n (%)	P-value (Chi-square)
**Child level factors**						
**Child age**			<0.001 (31.40)			<0.001 (51.29)
6–11 months	241 (12.7)	53 (8.4)		234 (11.9)	32 (7.2)	
12–23 months	432 (22.9)	139 (23.7)		467 (23.8)	143 (28.4)	
24–35 months	433 (22.9)	173 (29.0)		468 (23.8)	161 (31.6)	
36–47 months	396 (20.9)	129 (22.1)		410 (20.9)	104 (19.7)	
48–59 months	389 (20.6)	99 (16.7)		384 (19.6)	72 (13.1)	
**Child’s sex**			0.068 (3.33)			<0.001 (15.61)
Male	898 (47.5)	300 (51.3)		976 (49.7)	293 (58.2)	
Female	993 (52.5)	293 (48.7)		987 (50.3)	219 (41.8)	
**Maternal level factors**						
**Maternal age**			0.576 (4.75)			0.390 (6.30)
15–19	102 (5.4)	31 (51.2)		100 (5.1)	31 (5.5)	
20–24	481 (25.4)	160 (26.6)		466 (23.7)	116 (23.4)	
25–29	470 (24.9)	133 (22.9)		496 (25.3)	140 (28.5)	
30–34	294 (15.6)	87 (14.5)		370 (18.9)	87 (18.0)	
35–39	234 (12.4)	80 (12.8)		246 (12.5)	70 (12.4)	
40–44	192 (10.2)	65 (11.3)		175 (8.9)	45 (8.2)	
45–49	118 (6.2)	37 (6.5)		110 (5.6)	23 (3.9)	
**Maternal education**			<0.001 (68.27)			<0.001 (59.53)
None	150 (7.9)	61 (10.2)		126 (6.4)	45 (8.0)	
Primary	597 (31.6)	235 (40.9)		567 (28.9)	181 (35.3)	
Secondary	574 (30.4)	173 (28.7)		686 (35.0)	193 (37.1)	
High school	426 (22.5)	114 (18.7)		448 (22.8)	86 (18.4)	
Tertiary	144 (7.6)	10 (1.5)		136 (6.9)	7 (1.2)	
**Parity**			0.005 (11.53)			0.019 (7.90)
Less than 3	817 (43.2)	228 (37.7)		906 (46.2)	213 (44.4)	
3–4	561 (29.7)	178 (30.9)		616 (31.4)	164 (30.0)	
5 and above	513 (27.1)	187 (31.5)		441 (22.5)	135 (25.6)	
**Marital status**			0.016 (8.27)			0.195 (3.27)
Married	1136 (60.1)	337 (57.6)		1196 (60.9)	310 (61.4)	
Formerly married	173 (9.2)	70 (12.0)		167 (8.5)	53 (9.5)	
Never married	582 (30.8)	186 (30.4)		600 (30.5)	149 (29.1)	
**Household level factors**						
**Household wealth index**			<0.001 (81.05)			<0.001 (48.30)
Poorest	441 (23.3)	191 (32.0)		507 (25.8)	156 (25.4)	
Poor	363 (19.2)	120 (21.3)		480 (24.5)	153 (30.5)	
Middle	384 (20.3)	135 (23.0)		415 (21.1)	107 (21.4)	
Rich	348 (18.4)	95 (15.9)		304 (15.5)	69 (16.7)	
Richest	355 (18.8)	52 (7.8)		257 (13.1)	27 (6.1)	
**Source of drinking water**			<0.001 (13.37)			0.020 (5.45)
Improved	1262 (66.7)	361 (0.6)		1268 (64.6)	203 (0.3)	
Not improved	629 (33.3)	232 (0.4)		695 (35.4)	309 (0.7)	
**Household electricity**			<0.001 (50.89)			<0.001 (15.48)
Yes	1262 (66.7)	163 (26.6)		1036 (52.8)	232 (47.3)	
No	629 (33.3)	430 (73.4)		927 (47.2)	280 (52.7)	
**Toilet facility**			<0.001 (60.38)			<0.001 (32..79)
Flush toilet	272 (14.4)	34 (4.7)		256 (13.0)	35 (8.1)	
Pit latrine	1262 (66.7)	414 (71.3)		1400 (71.3)	370 (70.1)	
No facility, Bush, Field	357 (18.9)	145 (24.0)		307 (15.6)	107 (19.5)	
**Number of children under five**			<0.001 (19.59.)			<0.001 (20.63)
1	871 (46.1)	229 (37.2)		939 (47.8)	203 (42.3)	
2–3	882 (46.6)	312 (54.1)		906 (46.2)	267 (49.9)	
4 and more	138 (7.3)	52 (8.7)		118 (6.0)	42 (7.8)	
**Community-level factors**						
**Area of residence**			<0.001 (16.22)			0.012 (6.38)
Rural	1384 (73.2)	470 (83.7)		1615 (82.3)	440 (80.7)	
Urban	507 (26.8)	123 (16.3)		348 (17.7)	72 (19.3)	
**Region of residence**			0.012 (10.89)			0.622 (1.77)
Hhohho	430 (22.7)	126 (24.3)		478 (24.4)	114 (20.2)	
Manzini	483 (25.5)	137 (27.4)		531 (27.1)	140 (37.8)	
Shiselweni	504 (26.7)	187 (28.3)		516 (26.3)	138 (19.2)	
Lubombo	474 (35.1)	143 (19.9)		438 (22.3)	120 (22.8)	
**Community Poverty**			<0.001 (26.41)			<0.001 (16.89)
Low	665 (35.2)	160 (24.3)		662 (33.7)	144 (34.2)	
Medium	615 (32.5)	209 (37.7)		697 (35.5)	176 (31.3)	
High	611 (32.3)	224 (38.3)		604 (30.8)	192 (34.5)	
**Proportion of mothers with lower than secondary education level**			<0.001 (32.96)			<0.001 (19.44)
Low	684 (36.2)	159 (26.2)		703 (35.8)	144 (34.2)	
Medium	577 (30.5)	204 (33.8)		607 (30.9)	176 (31.3	
High	630 (33.3)	230 (40.0)		653 (33.3)	192(34.5)	

**Fig 1:**
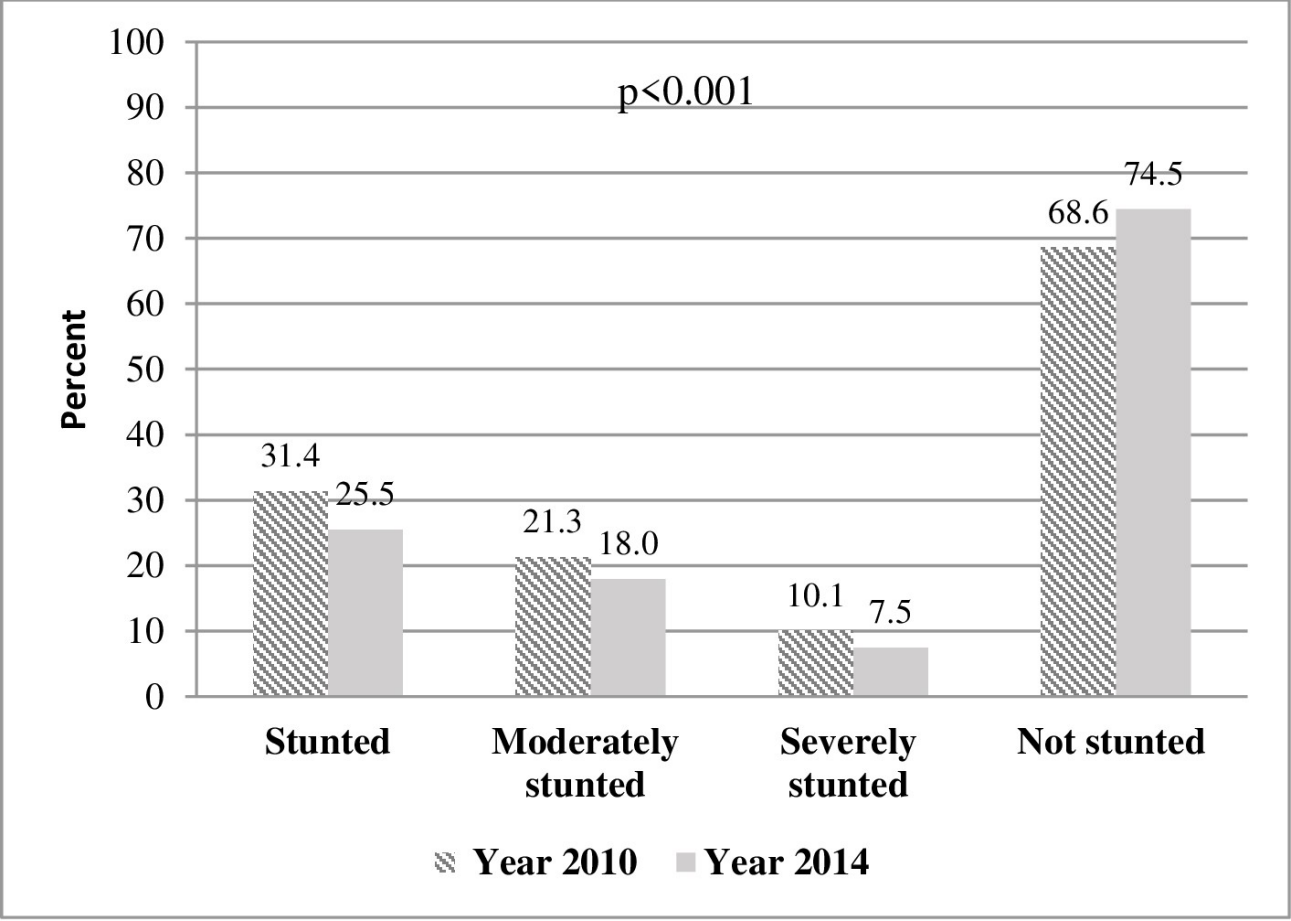
Prevalence of stunting among children aged 6–59 months.

There was a significant reduction in the prevalence of child stunting from 31.4%, (95% CI: 29.2, 33.6) in 2010 to 25.5%, (95% CI: 23.4, 27.7) in 2014, (p<0.001), the prevalence of moderate stunting from 21.3% to 18.0% and severe stunting from 10.1% to 7.5% as shown in Fig 1.

Table 1 shows the findings by explanatory factors in each survey year. In both surveys, the proportion of stunted children was lower among children aged 6–11 and 48–59 months. In 2010 there was no significant difference in stunting by child’s sex, whereas stunting was significantly higher among males versus females in 2014 (58.2% versus 41.8%), p<0.001. In both surveys, the proportion of stunted children significantly differed by maternal education status (p<0.001 in both survey years). In 2010, stunting varied by mothers’ marital status: married (57.6%), never-married (30.4%), and formerly married (12%) (p<0.05). The richest households had a significantly lower proportion of stunted children versus the poorest in both surveys (7.8% vs. 32% in 2010), p<0.001, and (6.1% vs. 25.4% in 2014), p<0.001. In 2010, the majority of stunted children were resident in the Shiselweni (28.3%) and Manzini (27.4%) regions (p<0.05). In 2014, however, there was no significant difference by region of residence. In both surveys, stunting significantly varied by parity, source of drinking water, household electricity, type of toilet facility, number of under five children in the household, area of residence, community poverty, and the proportion of mothers with lower than secondary education level in the community, (p<0.05).

### Multilevel analysis

The random effects and fixed effects results are shown in Table 2 for the 2010 survey and Table 3 for the 2014 survey. At the individual level, even after controlling for household and community level factors in both surveys, the children’s age was associated with stunting. In 2010, children aged 12–23 months, 24–35 and 36–47 months were more likely to be stunted, (AOR = 1.87, 95% CI: 1.25, 2.78), (AOR = 2.95, 95% CI: 1.97, 4.40) and (AOR = 1.88, 95% CI: 1.25, 2.82) respectively, as compared to children aged 6–11 months. In 2014, children aged 12–23 months were more likely to be stunted, (AOR = 3.19, 95% CI: 2.04, 5.00), 24–35 months (AOR = 3.81, 95% CI: 2.43, 5.96) and 36–47 months (AOR = 2.48, 95% CI: 1.56, 3.96) compared to those aged 6–11 months, holding other factors constant in the model. After adjusting for household and community level factors, in 2010, female children were 20% less likely to be stunted (AOR = 0.80, 95% CI: 0.64, 0.98) compared to male children. In 2014, female children were 33% less likely to be stunted (AOR = 0.67, 95% CI: 0.54, 0.84) compared to their male counterparts. In 2010, after controlling for household and community level factors, the estimates showed that children born to women with tertiary education were 78% less likely to be stunted (AOR = 0.22, 95% CI: 0.09, 0.50) compared to those born to women with no formal education. In 2014, children born to women with tertiary education were 82% less likely to be stunted (AOR = 0.18, 95% CI: 0.07, 0.46) compared to those born to women with no formal education.

**Table 2:** Results of the individual, household and community-level factors associated with child stunting, Eswatini MICS 2010.

Variables	Model 1	Model 2	Model 3	Model 4	Model 5
Fixed effects		AOR(95%CI)	AOR(95%CI)	AOR(95%CI)	AOR(95%CI)
**Child level factors**					
**Child age**					
6–11 months		1			1
12–23 months		1.77 (1.20, 2.27)*			1.87 (1.25, 2.78)*
24–35 months		2.64 (1.78, 3.91)*			2.95 (1.97, 4.40)*
36–47 months		1.81 (1.21, 2.70)*			1.88 (1.25, 2.82)*
48–59 months		1.25 (0.83, 1.89)			1.24 (0.82, 1.81)
**Sex**					
Male		1			1
Female		0.81 (0.66, 1.00)			0.80 (0.64, 0.98)*
**Maternal level factors**					
**Maternal age**					
15–19		1			1
20–24		1.22 (0.74, 2.03)			1.06 (0.64, 1.78)
25–29		1.07 (0.70, 2.32)			1.02 (0.59, 1.76)
30–34		1.27 (0.62, 1.83)			1.36 (0.74, 2.49)
35–39		1.24 (0.68, 2.25)			1.18 (0.62, 2.25)
40–44		1.14 (0.60, 2.14)			1.14 (0.58, 2.25)
45–49		0.76 (0.37, 1.58)			0.81 (0.39, 1.70)
**Maternal education**					
None		1			1
Primary		0.97 (0.65, 1.46)			0.94 (0.62, 1.42)
Secondary		0.61 (0.41, 0.93)*			0.70 (0.44, 1.10)
Higher		0.54 (0.34, 0.85)*			0.70 (0.43, 1.15)
Tertiary		0.11 (0.05, 0.23)*			0.22 (0.09, 0.50)*
**Parity**					
Less than 3		1			1
3–4		1.13 (0.84, 1.51)			1.02 (0.75, 1.37)
5 and above		1.24 (0.85, 1.82)			1.01 (0.68, 1.51)
**Marital status**					
Married		1			1
Formerly married		1.50 (1.04, 2.17)			1.34 (0.92, 1.95)
Never married		1.33 (1.02, 1.74)*			1.19 (0.91,1.57)
**Household level factors**					
**Household wealth index **					
Poorest			1		1
Poor			0.65 (0.47, 0.90)*		0.63 (0.45, 0.88)*
Middle			0.74 (0.53, 1.04)		0.76 (0.52, 1.11)
Rich			0.63 (0.53, 1.06)		0.66 (0.37, 1.17)
Richest			0.45 (0.23, 0.89)*		0.43 (0.25, 1.16)
**Source of drinking water **					
Improved			1		1
Not improved			1.03 (0.81, 1.30)		1.03 (0.81, 1.33)
**Household electricity**					
Yes			1		1
No			1.22(0.81, 1.82)		1.20 (0.79, 1.82)
**Toilet facility**					
Flush toilet			1		1
Pit latrine			2.02 (1.19, 3.44)*		1.87 (1.08, 3.26)*
No facility, Bush, Field			2.15 (1.16, 3.96)*		1.98 (1.04, 3.77)*
**Number of under-five children in the household**					
1			1		1
2–3			1.38 (1.11, 1.73)*		1.53 (1.21, 1.93)*
4 and more			1.38 (0.92, 2.09)		1.59 (1.03, 2.45)*
**Community Level factors **					
**Area of residence **					
Rural				0.94 (0.65, 1.36)	0.68 (0.46, 1.02)
Urban				1	1
**Region of residence**					
Hhohho				1.21 (0.86, 1.70)	1.18 (0.83, 1.70)
Manzini				1.23 (0.87, 1.73)	1.17 (0.82, 1.68)
Shiselweni				1.47 (1.07, 2.02)*	1.42 (1.02, 1.98)*
Lubombo				1	1
**Community Poverty**					
Low				1	1
Medium				1.42 (1.01, 2.03)	0.97 (0.66, 1.42)
High				1.49 (1.02, 2.19)	0.92 (0.59, 1.75)
**Proportion of mothers with lower than secondary education level in the community**					
Low				1	1
Medium				1.71 (1.26, 2.32)*	1.40 (1.01, 1.94)
High				1.75 (1.27, 2.41)*	1.21 (0.84, 1.75)
**Random effects**	**Empty**	**Individual**	**Household**	**Community**	**Final model**
Community variance (SE)	0.284 (0.102)*	0.209 (0.095)*	0.182 (0.091)*	0.183 (0.088)*	0.169 (0.092)*
VPC = ICC (%)	8.0	6.0	5.3	5.7	4.9
PCV (%)	Reference	79.0	81.6	35.8	40.6
Log likelihood	-1169.30	-1108.01	-1121.76	-1148.23	-1078.99
Observations	1,891	1,891	1,891	1,891	1,891
AIC	23421.60	2258.02	2267.53	2316.47	2235.98

*significant at p<0.05, SE-standard error, ICC-intraclass correlation coefficient, PVC-proportion change in variance, AOR-adjusted odds ratio, AIC-Akaike information criterion

**Table 3:** Results of the individual, household and community-level factors associated with child stunting, Eswatini MICS 2014.

Variables	Model 1	Model 2	Model 3	Model 4	Model 5
Fixed effects		AOR (95%CI)	AOR (95%CI)	AOR(95%CI)	AOR (95%CI)
**Child level factors**					
**Child age**					
6–11 months		1			1
12–23 months		3.09 (1.98, 4.82)*			3.19 (2.04, 5.00)*
24–35 months		3.76 (2.41, 5.87)*			3.81 (2.43, 5.96)*
36–47 months		2.43 (1.53, 3.85)*			2.48 (1.56, 3.96)*
48–59 months		1.62 (1.00, 2.61)			1.58 (0.97, 2.57)
**Sex**					
Male		1			1
Female		0.67 (0.54, 0.83)*			0.67 (0.54,0.84)*
**Maternal level factors**					
**Maternal age**					
15–19		1			1
20–24		0.76 (0.45, 1.29)			0.77 (0.45, 1.31)
25–29		0.89 (0.51, 1.54)			0.94 (0.54, 1.66)
30–34		0.64 (0.35, 1.18)			0.77 (0.41, 1.45)
35–39		0.74 (0.38, 1.42)			0.93 (0.47, 1.82)
40–44		0.56 (0.28, 1.12)			0.71 (0.35, 1.45)
45–49		0.39 (0.18, 0.84)*			0.45 (0.21, 1.01)
**Maternal education**				
None		1			1
Primary		0.78 (0.50, 1.23)			0.79 (0.50, 1.25)
Secondary		0.68 (0.44, 1.07)			0.76 (0.47, 1.22)
Higher		0.41 (0.25, 0.67)*			0.49 (0.29, 0.84)*
Tertiary		0.10 (0.04, 0.25)*			0.18 (0.07, 0.46)*
**Parity**					
Less than 3		1			1
3–4		1.17 (0.86, 1.62)			1.07 (0.78, 1.48)
5 and above		1.50 (0.99, 2.26)			1.18 (0.77, 1.80)
**Marital status**					
Married		1			1
Formerly married		1.28 (0.87, 1.88)			1.14 (0.77, 1.48)
Never married		1.07 (0.81, 1.41)			1.18 (0.77, 1.80)
**Household level factors**					
**Household wealth index**				
Poorest			1		1
Poor			1.17 (0.85, 1.62)		1.16 (0.82, 1.64)
Middle			0.92 (0.62, 1.37)		0.87 (0.55, 1.37)
Rich			0.83 (0.51, 1.36)		0.82 (0.46, 1.47)
Richest			0.37 (0.19, 0.79)*		0.45 (0.21, 0.96)*
**Source of drinking water**				
Improved			1		1
Not improved			0.98 (0.77, 1.25)		1.02 (0.79, 1.32)
**Household electricity**				
Yes			1		1
No			1.04 (0.77, 1.40)		0.99 (0.72, 1.37)
**Toilet facility**					
Flush toilet			1		1
Pit latrine			1.21 (0.75, 1.96)		1.12 (0.68, 1.87)
No facility, Bush, Field		1.65 (0.93, 2.94)		1.30 (0.70, 2.43)
**Number of under five children in the household**					
1			1		1
2–3			1.42 (1.14, 1.78)*		1.48 (1.16, 1.89)*
4 and more			1.69 (1.08, 2.65)*		1.74 (1.08, 2.81)*
**Community Level factors**					
**Area of residence**					
Rural				1.12 (0.78, 1.61)	0.80 (0.52, 1.21)
Urban				1	1
**Region of residence**					
Hhohho				1.05 (0.74, 1.50)	1.13 (0.77, 1.67)
Manzini				1.28 (0.90, 1.83)	1.32 (0.89, 1,96)
Shiselweni				1.19 (0.84, 1.68)	1.22 (0.83, 1.78)
Lubombo					1
**Community Poverty**					
Low				1	1
Medium				1.13 (0.81, 1.56)	0.85 (0.58, 1.23)
High				1.47 (1.03, 2.09)*	0.95 (0.61, 1.47)
**Proportion of mothers with lower than secondary education level in the community**					
Low				1	1
Medium				1.18 (0.86, 1.60)	1.06 (0.76, 1.48)
High				1.52 (1.11, 2.08)*	1.18 (0.82, 1.69)
**Random effects**	**Empty**	**Individual**	**Household**	**Community**	**Final model**
Community variance (SE)	0.260 (0.102)*	0.235 (0.106)*	0.201 (0.096)*	0.179 (0.092)*	0.212 (0.104)*
VPC = ICC (%)	7.3	6.7	5.8	5.2	6.1
PCV (%)	Reference	9.6	20.5	31.0	76.7
Log likelihood	-1121.09	-1047.38	-1084.55	-1109.38	-1028.0
Observations	1,963	1,963	1,963	1,963	1,963
AIC	2246.17	2136.77	2193.1	2238.75	2134.12

*significant at p<0.05, SE-standard error, ICC-intraclass correlation coefficient, PVC-proportion change in variance, AOR-adjusted odds ratio, AIC-Akaike information criterion

Controlling for all other factors in the model, children from households classified as rich were less likely to be stunted than those from the poorest households in both 2010 and 2014. For example, in 2014, the likelihood of being stunted was significantly lower for children from the richest households (AOR = 0.45, 95% CI: 0.21, 0.96) compared to those from the poorest households. In 2010, higher odds of being stunted were observed among children from households with a pit latrine (AOR = 1.87, 95% CI: 1.08, 3.26) and no toilet facility (AOR = 1.98, 95% CI: 1.04, 3.77), compared to those from households with a flush toilet. However, in 2014, there was no significant association between the toilet facility and stunting. In 2010, children from households with two to three (AOR = 1.53, 95% CI: 1.21, 1.93), and four and more children (AOR = 1.59, 95% CI: 1.03, 2.45) under five years of age had a higher likelihood of being stunted compared to those from households with only one child. Similarly, in 2014, higher odds of being stunted were observed among children from households with two to three children (AOR = 1.48, 95% CI: 1.16, 1.89), and four and more children under five years of age (AOR = 1.74, 95% CI: 1.08, 2.81) compared to those from households with only one child. Regionally, in 2010, children from the Shiselweni were 42% more likely to be stunted (AOR = 1.42, 95% CI: 1.02, 1.98) compared to those from the Lubombo region. In 2014, the region of residence was not significantly associated with stunting.

The analysis also showed the random effect estimates. The empty model, the intercept only model was fitted to justify if there was random effect at the community level. In Table 2, empty model (model 1) there was a significant variation in the odds of child stunting (τ = 0.284, p<0.001 in 2010, τ = 0.260, p<0.001 in 2014). The intraclass correlation coefficient (ICC) revealed that 8% and 7.3% in 2010 and 2014, respectively of the variation of child stunting could be attributed to the difference in the composition of the communities. The variance remained significant even after controlling for individual, household and community level factors (model 5). Tables 2 and 3 further show the model fit statistics. In both survey years, the Akaike Information Criterion (AIC) estimates showed significant reduction indicating that the model improves, revealing a better fit for the data. Therefore, after combining individual, household and community level factors in one model the model specification improved compared to the other models. The combined model (model 5) was therefore chosen to predict child stunting.

## Discussions

The study found a slight significant decrease in the prevalence of child stunting between 2010 and 2014, and several factors were associated with the reduction in stunting in the two survey periods. The magnitude of child stunting is relatively low in Eswatini when compared to that of Zambia (40%) [10], Mozambique (51%) [34] and Malawi (48.4%) [30]. The decline in stunting is in line with the global agenda to achieve SDG goal number two, to end hunger and improve nutrition by 2030 [6, 35]. The observed decrease in child stunting may have been a result of programs spearheaded by the Eswatini Nutrition Council and UNICEF to identify stunted children at the community and facility level for proper referral and management at a health facility [36]. Other programs implemented include educational programs focusing on females [26], family planning and access and use of modern contraceptives [19] and national efforts aimed at improving access to safe water in communities and poverty alleviation. i.e in 2012 the poverty rate decreased to 63% from 69% in year 2000 [37, 38]. However, stunting is still a significant public health challenge in Eswatini.

The study found that children aged 12–23, 24–35, and 36–47 months had higher odds of being stunted compared to those aged 6–11 months. The study findings are consistent with other studies that the odds of stunting increased from age 12–47 months [15, 16]. This study found lower odds of being stunted among female children. Other studies done in developing countries also reported a lower likelihood of being stunted among female children compared to their male counterparts [10, 16]. A multicounty analysis of 16 Demographic and Health Surveys from 10 developing countries reported similar findings that female children were less likely to be stunted [39]. This could be due to child feeding preferences or other health exposures and that male children are biologically more predisposed to sicknesses compared to females [13, 40, 41].

This study found that an increase in mothers’ education was associated with lower odds of child stunting. Other studies done in Zambia, Nigeria, and Rwanda reported a lower risk of child stunting among children born to educated mothers [10, 17, 42]. The care afforded by the mother to the child, including access to medical care and proper nutrition and hygiene, was influenced by the mother's educational level [43]. The reduced odds of stunting among children from the richest households were also observed in other studies [13, 44, 45].

The results showed that children from households that used a pit latrine or did not have a toilet facility at all had increased odds of being stunted. Similarly, an analysis of 137 Demographic and Health surveys in developing countries showed that children with access to improved sanitation had reduced odds of stunting among children under five years [46]. In line with the literature [14, 47, 48], this study found that children from households with two or more children aged under five years were more likely to be stunted.

Studies have reported variations in child stunting by region of residence [15, 16, 31]. The prevalence of stunting was lower by 9.1 points between 2010 and 2014 in the Shiselweni region between 2010 and 2014 (see Table 1). An explanation for this finding could be due to the impact of programs implemented in the Shiselweni region by organisations such as Save the children and World Vision in giving food hampers to needy children and households to improve their nutrition. Children that were residents in the Shiselweni region had higher odds of being stunted in 2010, although in 2014 there was no significant variation between the regions. Even though the poverty incidence reduced from 82% in 2001 to 68% in 2010 in the Shiselweni region, it was still the impoverished region relative to the other regions [25, 49].

### Study strengths and limitations

This study should be interpreted with caution. It was cross-sectional; hence it was challenging to establish a sequence of events and causal inference between stunting and individual, household, and community-level factors. The use of secondary data is a limitation due to the limited number of variables available in MICSs yet important for explaining child stunting, such as dietary intake and health care-related factors. Recall bias, and misreporting some information, such as on children age and birth size, cannot be ruled out in the EMICS. The study aggregated the community-level factors at the enumeration area (EA); this may have resulted in some children being misclassified. This study can only be interpreted in the context of Eswatini. Regardless of the above limitations, the study has several strengths. Firstly, we utilized data from a nationally representative survey, which improves the generalizability of the findings. Secondly, a multilevel analysis was used due to the hierarchal nature of the EMICSs. Thirdly, we accounted for the multistage complex sampling design of the two surveys through weighting, which further increased the validity of the study.

## Conclusion

In summary, the study findings indicate a slight decrease of stunting in children 6–59 months of age between 2010 and 2014 in Eswatini. Even though stunting slightly reduced between 2010 and 2014, it remains a public health problem in Eswatini. Stunting was high among males versus female children and who have two or more children under five years in the household in both 2010 and 2014. In 2010 stunting was higher among children from households with a pit latrine or no toilet facility versus those from households with a flush toilet facility and from the Shiselweni region. In both 2010 and 2014 child stunting was significantly lower amongst children whose mother had received tertiary education while in 2014 stunting was significantly lower among children from rich households. Overall, a multisectoral program that targets integrated interventions at the individual, household, and community factors could yield positive gains in the reduction of child stunting to achieve the SGDs by 2030.

## Supporting information

S1 File Eswatini MICS questionnaire for children under five years. (DOC)

## References

[1] Liu L, Oza S, Hogan D, Chu Y, Perin J, Zhu J, et al Global, regional, and national causes of under-5 mortality in 2000–15: an updated systematic analysis with implications for the Sustainable Development Goals. Lancet (London, England). 2016;388(10063):3027–35. 10.1016/S0140-6736(16)31593-8 PMC5161777 27839855

[2] Mejía-Guevara I, Zuo W, Bendavid E, Li N, Tuljapurkar S. Age distribution, trends, and forecasts of under-5 mortality in 31 sub-Saharan African countries: A modeling study. PLoS medicine. 2019;16(3):e1002757 10.1371/journal.pmed.1002757 30861006 PMC6413894

[3] Wasting-Stunting Technical Interest Group (WaSt TIG). Child wasting and stunting: Time to overcome the separation. www.ennonline.net/resources/timetoovercometheseparation. 2018.

[4] World Health Organization (WHO). Nutrition Landscape Information System (NLIS) country profile indicators: Interpretation guide. Geneva, Switzerland: WHO, 2010.

[5] World Health Organization. Global Nutrition Monitoring Framework: operational guidance for tracking progress in meeting targets for 2025. World Health Organization, 2017.

[6] UN General Assembly. Transforming our world: the 2030 Agenda for Sustainable Development, 2015 [available at: https://www.refworld.org/docid/57b6e3e44.html, [accessed 13 August 2019].

[7] Central Statistical Office, UNICEF. Eswatini Multiple Indicator Cluster Survey 2014. Final Report,. Mbabane, Eswatini, Central Statistical Office and UNICEF,: 2016.

[8] Central Statistical Office, UNICEF. Eswatini Multiple Indicator Cluster Survey 2010. Final Report. Mbabane, Eswatini,: Central Statistical Office and UNICEF, 2011.

[9] Nshimyiryo A, Hedt-Gauthier B, Mutaganzwa C, Kirk CM, Beck K, Ndayisaba A, et al Risk factors for stunting among children under five years: a cross-sectional population-based study in Rwanda using the 2015 Demographic and Health Survey. BMC Public Health. 2019;19(1):175 10.1186/s12889-019-6504-z 30744614 PMC6371425

[10] Mzumara B, Bwembya P, Halwiindi H, Mugode R, Banda J. Factors associated with stunting among children below five years of age in Zambia: evidence from the 2014 Zambia demographic and health survey. BMC Nutrition. 2018;4(1):51 10.1186/s40795-018-0260-9 32153912 PMC7050779

[11] Buisman LR, Van de Poel E, O'Donnell O, van Doorslaer EKA. What explains the fall in child stunting in Sub-Saharan Africa? SSM—population health. 2019;8:100384 10.1016/j.ssmph.2019.100384 31193968 PMC6545382

[12] Kismul H, Acharya P, Mapatano MA, Hatløy A. Determinants of childhood stunting in the Democratic Republic of Congo: further analysis of Demographic and Health Survey 2013–14. BMC public health. 2017;18(1):74 10.1186/s12889-017-4621-0 28764669 PMC5540220

[13] Chirande L, Charwe D, Mbwana H, Victor R, Kimboka S, Issaka AI, et al Determinants of stunting and severe stunting among under-fives in Tanzania: evidence from the 2010 cross-sectional household survey. BMC pediatrics. 2015;15:165 10.1186/s12887-015-0482-9 26489405 PMC4618754

[14] Darteh EKM, Acquah E, Kumi-Kyereme A. Correlates of stunting among children in Ghana. BMC Public Health. 2014;14(1):504 10.1186/1471-2458-14-504 24884653 PMC4049451

[15] Fantay Gebru K, Mekonnen Haileselassie W, Haftom Temesgen A, Oumer Seid A, Afework Mulugeta B. Determinants of stunting among under-five children in Ethiopia: a multilevel mixed-effects analysis of 2016 Ethiopian demographic and health survey data. BMC pediatrics. 2019;19(1):176 10.1186/s12887-019-1545-0 31153381 PMC6544992

[16] Haile D, Azage M, Mola T, Rainey R. Exploring spatial variations and factors associated with childhood stunting in Ethiopia: spatial and multilevel analysis. BMC pediatrics. 2016;16:49 10.1186/s12887-016-0587-9 27084512 PMC4833938

[17] Adekanmbi VT, Kayode GA, Uthman OA. Individual and contextual factors associated with childhood stunting in Nigeria: a multilevel analysis. Maternal & Child Nutrition. 2013;9(2):244–59. 10.1111/j.1740-8709.2011.00361.x 22004134 PMC6860873

[18] Central Statistical Office. The 2017 population and housing census preliminary report. Mbabane, Swaziland: The Government of the Kingdom of Swaziland and UNFPA, 2017.

[19] United Nation Population Fund (UNFPA). Renewing Commitments on the three zeros. Mbabane, Eswatini: UNFPA. Accessed 15 September 2020 from https://eswatini.unfpa.org/sites/default/files/pub- pdf/unfpa_eswatini_annual_report_2019.pdf, 2019.

[20] Central Statitsics Office. Swaziland Household Income and Exprenditure survey 2016/2017, preliminary findings. Mbabane, Swaziland: 2018.

[21] UNICEF. Demographic, Health and infant mortality. Accessed 11 September 2020 from https://data.unicef.org/country/swz/.

[22] UNICEF. UNICEF Annual Report 2017. Accessed 14 Septemeber 2020 from https://www.unicef.org/about/annualreport/files/Swaziland_2017_COAR.pdf. 2017.

[23] WHO. Antenatal Care. https://www.who.int/pmnch/media/publications/aonsectionIII_2.pdf 2016.

[24] Tsawe M, Moto A, Netshivhera T, Ralesego L, Nyathi C, Susuman AS. Factors influencing the use of maternal healthcare services and childhood immunization in Swaziland. International Journal for Equity in Health. 2015;14(1):32 10.1186/s12939-015-0162-2 25889973 PMC4391603

[25] Eswatini Ministry of Agriculture. Cash transfer intervention modality options Mbabane, Eswatini,: 2016.

[26] Eswatini Ministry of Education and Training. The Swaziland Education for all review report, 2000–2015,. Mbabane, Eswatini. Accessed 15 September 2020 from https://unesdoc.unesco.org/ark:/48223/pf0000232703: 2015.

[27] UNICEF. Swaziland Humanitarian Situation Report. Accessed 14 September 2020 from https://www.unicef.org/appeals/files/UNICEF_Swaziland_Humanitarian_SitRep_30_April_2017.pdf. 2017.

[28] Simelane MS, Shongwe MC, Vermaak K, Zwane E. Determinants of Households’ Access to Improved Drinking Water Sources: A Secondary Analysis of Eswatini 2010 and 2014 Multiple Indicator Cluster Surveys. Advances in Public Health. 2020;2020:6758513.

[29] Titaley CR, Ariawan I, Hapsari D, Muasyaroh A, Dibley MJ. Determinants of the Stunting of Children Under Two Years Old in Indonesia: A Multilevel Analysis of the 2013 Indonesia Basic Health Survey. Nutrients. 2019;11(5):1106 10.3390/nu11051106 31109058 PMC6567198

[30] Ntenda PAM, Chuang Y-C. Analysis of individual-level and community-level effects on childhood undernutrition in Malawi. Pediatrics & Neonatology. 2018;59(4):380–9. 10.1016/j.pedneo.2017.11.019 29295806

[31] Adekanmbi VT, Uthman OA, Mudasiru OM. Exploring variations in childhood stunting in Nigeria using league table, control chart and spatial analysis. BMC Public Health. 2013;13(1):361 10.1186/1471-2458-13-361 23597167 PMC3640947

[32] Wit JM, Himes JH, van Buuren S, Denno DM, Suchdev PS. Practical Application of Linear Growth Measurements in Clinical Research in Low- and Middle-Income Countries. Hormone Research in Paediatrics. 2017;88(1):79–90. 10.1159/000456007 28196362 PMC5804842

[33] Raykov T. Intraclass Correlation Coefficients in Hierarchical Designs: Evaluation Using Latent Variable Modeling. Structural Equation Modeling: A Multidisciplinary Journal. 2011;18(1):73–90.

[34] Rose ES, Blevins M, González-Calvo L, Ndatimana E, Green AF, Lopez M, et al Determinants of undernutrition among children aged 6 to 59 months in rural Zambézia Province, Mozambique: results of two population-based serial cross-sectional surveys. BMC Nutrition. 2015;1(1):41 10.1186/s40795-015-0039-1 27182448 PMC4864006

[35] UNDP. Human Development Indices and Indicators: 2018 Statistical Update.Briefing note for countries on the 2018 Statistical Update, Kingdom of Eswatini, 2018.

[36] UNICEF. UNICEF annual report Mbabane, Eswatini,: 2017.

[37] Kunene S. ‘Lived poverty’ can inform Swazi anti-poverty efforts. Afrobarometer Briefing Paper No. 155. https://media.africaportal.org/documents/Afrobriefno155_1.pdf, 2015.

[38] Eswatini Government. Country Nutrition Paper;Joint FAO/WHO Second International Conference on Nutrition ICN2. Second International Conference on Nutrition Rome, Italy2014.

[39] Wamani H, Åstrøm AN, Peterson S, Tumwine JK, Tylleskär T. Boys are more stunted than girls in Sub-Saharan Africa: a meta-analysis of 16 demographic and health surveys. BMC Pediatrics. 2007;7(1):17 10.1186/1471-2431-7-17 17425787 PMC1865375

[40] Elsmén E, Pupp IH, Hellström-Westas L. Preterm male infants need more initial respiratory and circulatory support than female infants. Acta Paediatrica. 2004;93(4):529–33. 10.1080/08035250410024998 15188982

[41] Condo JU, Gage A, Mock N, Rice J, Greiner T. Sex differences in nutritional status of HIV-exposed children in Rwanda: a longitudinal study. Tropical Medicine & International Health. 2015;20(1):17–23. 10.1111/tmi.12406 25345559

[42] Nyiraneza L, Wong R, Olu O, Nahimana M-R, Birachi E, Musoni A, et al Risk Factors Associated With Childhood Stunting in Rwanda: A Secondary Analysis of the 2014 Nutrition, Markets and Gender (NMG) Survey. Journal of Management and Strategy. 2019;10:34.

[43] Menezes RCEd Lira PICd, Leal VS Oliveira JS, Santana SCdS Sequeira LAdS, et al Determinantes do déficit estatural em menores de cinco anos no Estado de Pernambuco. Revista de Saúde Pública. 2011;45:1079–87. 10.1590/s0034-89102011000600010 22124738

[44] Urke HB, Bull T, Mittelmark MB. Socioeconomic status and chronic child malnutrition: wealth and maternal education matter more in the Peruvian Andes than nationally. Nutrition Research. 2011;31(10):741–7. 10.1016/j.nutres.2011.09.007 22074798

[45] Akombi BJ, Agho KE, Hall JJ, Wali N, Renzaho AMN, Merom D. Stunting, Wasting and Underweight in Sub-Saharan Africa: A Systematic Review. International journal of environmental research and public health. 2017;14(8):863 10.3390/ijerph14080863 28788108 PMC5580567

[46] Danaei G, Andrews KG, Sudfeld CR, Fink G, McCoy DC, Peet E. Risk Factors for Childhood Stunting in 137 Developing Countries: A Comparative Risk Assessment Analysis at Global, Regional, and Country Levels. 2016;13(11):e1002164 10.1371/journal.pmed.1002164 27802277 PMC5089547

[47] Cruz L, Azpeitia G, Súarez D, Rodríguez A, Loro J, Serra-Majem L. Factors Associated with Stunting among Children Aged 0 to 59 Months from the Central Region of Mozambique. Nutrients. 2017;9:491 10.3390/nu9050491 28498315 PMC5452221

[48] Ramos CV, Dumith SC, César JA. Prevalence and factors associated with stunting and excess weight in children aged 0–5 years from the Brazilian semi-arid region. Jornal de Pediatria. 2015;91:175–82. 10.1016/j.jped.2014.07.005 25449789

[49] Central Statistics Office. Poverty in a decade of slow economic growth:Swaziland in the 2000 's Mbabane, Eswatini. 2011.

