# Supplementary material for: A multilevel analysis of individual, household and community level factors on stunting among children aged 6–59 months in Eswatini: A secondary analysis of the Eswatini 2010 and 2014 Multiple Indicator Cluster Surveys
Source: PLoS One. 2020 Oct 30;15(10):e0241548. doi: 10.1371/journal.pone.0241548 (PMC7598515; doi:10.1371/journal.pone.0241548)
Supplement: S1 File — (DOC) [file pone.0241548.s001.doc]

| 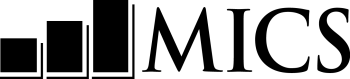questionnaire for children under five  *Swaziland* | |
| --- | --- |
|  | |
| under-five child information panel uf | |
| This questionnaire is to be administered to all mothers or caretakers (see List of Household Members, column HL15) who care for a child that lives with them and is under the age of 5 years (see List of Household Members, column HL7B).  A separate questionnaire should be used for each eligible child. | |
| **UF1**. Cluster number | **UF2**. Household number: |
| ___ ___ ___ | ___ ___ |
| **UF3**. Child’s name: | **UF4**. Child’s line number: |
| Name | ___ ___ |
| **UF5**. Mother’s / Caretaker’s name: | **UF6**. Mother’s / Caretaker’s line number: |
| Name | ___ ___ |
| **UF7**. Interviewer’s name and number: | **UF8**. Day / Month / Year of interview: |
| Name ___ ___ | ___ ___ /___ ___ / 2 0 14 |

| Repeat greeting if not already read to this respondent:  We are from Central Statistical Office. We are conducting a survey about the situation of children, families and households. I would like to talk to you about (*child’s name from UF3*)’s health and well-being. The interview will take about 15 minutes. All the information we obtain will remain strictly confidential and anonymous. | *If greeting at the beginning of the household questionnaire has already been read to this person, then read the following:*  Now I would like to talk to you more about (*child’s name from UF3*)’s health and other topics. This interview will take about 15 minutes. Again, all the information we obtain will remain strictly confidential and anonymous. |
| --- | --- |
| May I start now?   - Yes, permission is given ⇨ *Go to UF12 to record the time and then b*egin the interview. - No, permission is not given ⇨ Circle ‘03’ in UF9. Discuss this result with your supervisor. | |

| **UF9**. Result of interview for children under 5  Codes refer to mother/caretaker. | Completed 01  Not at home 02  Refused 03  Partly completed 04  Incapacitated 05  Other (*specify*) 96 |
| --- | --- |

| **UF10**. Field editor’s name and number:  Name______________________________ __ __ | **UF11**. Main data entry clerk’s name and number:  Name_______________________________ __ __ |
| --- | --- |

| **UF12**. *Record the time.* | Hour and minutes __ __ : __ __ |  |
| --- | --- | --- |

| age AG | | |
| --- | --- | --- |
| **AG1**. Now I would like to ask you some questions about the development and health of (*name*).  On what day, month and year was (name) born?  Probe:  What is his / her birthday?  If the mother/caretaker knows the exact birth date, also enter the day; otherwise, circle 98 for day.  Month and year must be recorded. | Date of birth  Day __ __  DK day 98  Month __ __  Year 2 0 __ __ |  |
| **AG2**. How old is (name)?  Probe:  How old was (name) at his / her last birthday?  Record age in completed years.  Record ‘0’ if less than 1 year.  Compare and correct AG1 and/or AG2 if inconsistent. | Age (in completed years) __ |  |

| birth registration BR | | |
| --- | --- | --- |
| **BR1**. Does (name) have a birth certificate?  *If yes, ask:*  May I see it? | Yes, seen 1  Yes, not seen 2  No 3  DK 8 | 3 ⇨ BR2  8 ⇨ BR2 |
| **BR1A**. Whose particulars appear on the birth certificate? | Mothers only 1  Fathers only 2  Both ………………………………………….. 3  DK/ Don't remember 8 | 1⇨Next  Module  2⇨Next  Module  3⇨Next  Module  8 ⇨Next  Module |
| **BR2.** Has **(*name*)’s** birth been registered with the civil authorities? | Yes 1  No 2  DK 8 | 1⇨Next  Module |
| **BR3**. Do you know how to register (name)’s birth? | Yes 1  No 2 | 2 ⇨Next  Module |
| **BR4**. What is the main reason for not registering (name)’s birth? | Registration costs too much 01  Offices too far (travel costs) 02  Did not know child should be registered 03  Did not want to pay fine 04  Partner refuses 05  No need to register child’s birth ............... 06  Father/ Mother does not have a PIN/ID 07  Other (*specify*)______________________96  DK 98 |  |

| early Childhood development eC | | |
| --- | --- | --- |
| **EC1**. How many children’s books or picture books do you have for (name)? | None 00  Number of children’s books 0 __  Ten or more books 10 |  |
| **EC2**. I am interested in learning about the things that (name) plays with when he/she is at home.  Does he/she play with:  [A] homemade toys (such as dolls, cars, or other toys made at home)?  [B] toys from a shop or manufactured toys?  [C] household objects (such as bowls or pots) or objects found outside (such as sticks, rocks, animal shells or leaves)?    If the respondent says “YES” to the categories above, then probe to learn specifically what the child plays with to ascertain the response. | Y N DK  Homemade toys 1 2 8  Toys from a shop 1 2 8  Household objects  or outside objects 1 2 8 |  |
| **EC3**. Sometimes adults taking care of children have to leave the house to go shopping, wash clothes, or for other reasons and have to leave young children.  On how many days in the past week was (name):  [A] left alone for more than an hour?  [B] left in the care of another child, that is, someone less than 10 years old, for more than an hour?  If ‘none’ enter’ 0’. If ‘don’t know’ enter’8’. | Number of days left alone for  more than an hour __  Number of days left with other  child for more than an hour __ |  |
| ***EC4****.* Check AG2: Age of child.  **🞎** Child age 0, 1 or 2 ⇨ Go to Next Module.  **🞎**  Child age 3 or 4 ⇨ Continue with EC5. | | |
| **EC5**. Does (name) attend any organized learning or early childhood education programme, such as a private or government facility, including kindergarten or community child care? | Yes 1  No 2  DK 8 | 1⇨EC5B |

| **EC5A.** What is the main reason (name) is not attending any organized learning or early childhood education programme, such as a private or government facility, including kindergarten or community child care? | Too far 1  Too costly 2  Disability 3  Religion 4  Other (specify)_______________________6  DK 8 | | | | | 1⇨EC7  2⇨EC7  3⇨EC7  4⇨EC7  6⇨EC7  8⇨EC7 |
| --- | --- | --- | --- | --- | --- | --- |
| **EC5B.** What type of organized learning or early childhood education programme, does (name) attend? | NCP/Community……………………………...1  Church…………………………………………2  Private…………………………………………3  NGO aided…………………………………….5  Other (specify)______________________ 6 | | | | |  |
| **EC6.** Within the last seven days, about how many hours did (name) attend? | Number of hours __ __ | | | | |  |
| **EC7**. In the past 3 days, did you or any household member age 15 or over engage in any of the following activities with (name):  If yes, ask:  Who engaged in this activity with (name)?  Circle all that apply. |  | | | | |  |
|  |  | Mother | Father | Other | No  one |  |
| [A] Read books to or looked at picture  books with (name)? | Read books | A | B | X | Y |  |
| [B] Told stories to (name)? | Told stories | A | B | X | Y |  |
| [C] Sang songs to (name) or with (name),  including lullabies? | Sang songs | A | B | X | Y |  |
| [D] Took (name) outside the home,  compound, yard or enclosure? | Took outside | A | B | X | Y |  |
| [E] Played with (name)? | Played with | A | B | X | Y |  |
| [F] Named, counted, or drew things  to or with (name)? | Named/counted | A | B | X | Y |  |
| **EC8**. I would like to ask you some questions about the health and development of (name). Children do not all develop and learn at the same rate. For example, some walk earlier than others. These questions are related to several aspects of (name)’s development.  Can (name) identify or name at least ten letters of the alphabet? | Yes 1  No 2  DK 8 | | | | |  |

| **EC9**. Can (name) read at least four simple, popular words? | Yes 1  No 2  DK 8 |  |
| --- | --- | --- |
| **EC10**. Does (name) know the name and recognize the symbol of all numbers from 1 to 10? | Yes 1  No 2  DK 8 |  |
| **EC11**. Can (name) pick up a small object with two fingers, like a stick or a rock from the ground? | Yes 1  No 2  DK 8 |  |
| **EC12**. Is (name) sometimes too sick to play? | Yes 1  No 2  DK 8 |  |
| **EC13**. Does (name) follow simple directions on how to do something correctly? | Yes 1  No 2  DK 8 |  |
| **EC14**. When given something to do, is (name) able to do it independently? | Yes 1  No 2  DK 8 |  |
| **EC15**. Does *(*name) get along well with other children? | Yes 1  No 2  DK 8 |  |
| **EC16**. Does (name) kick, bite, or hit other children or adults? | Yes 1  No 2  DK 8 |  |
| **EC17**. Does (name) get distracted easily? | Yes 1  No 2  DK 8 |  |

| breastfeeding and dietary intake Bd | | |
| --- | --- | --- |
| ***BD1.*** Check AG2: Age of child  **🞎**  Child age 0, 1 or 2 ⇨ Continue with BD2.  **🞎** Child age 3 or 4 ⇨ Go to Care of Illness Module. | | |
| **BD2**. Has (name) ever been breastfed? | Yes 1  No 2  DK 8 | 1⇨BD3  8⇨BD4 |
| **BD2A**. What is the main reason (name) has never been breastfed? | No milk 1  Child refused breastfeeding 2  Fear of infecting child................................ 3  Child too-ill to breastfeed……………..….…4  Mother too-ill to breastfeed………….……..5  Other (specify)_____________________6 | 1⇨BD4  2⇨BD4  3⇨BD4  4⇨BD4  5⇨BD4  6⇨BD4 |
| **BD3**. Is (name) still being breastfed? | Yes 1  No 2  DK 8 |  |
| **BD4**. Yesterday, during the day or night, did (name) drink anything from a bottle with a nipple? | Yes 1  No 2  DK 8 |  |
| **BD5**. Did (name) drink ORS (oral rehydration solution) yesterday, during the day or night? | Yes 1  No 2  DK 8 |  |
| **BD6**. Did (name) drink or eat vitamin or mineral supplements or any medicines yesterday, during the day or night? | Yes 1  No 2  DK 8 |  |

| **BD7**. Now I would like to ask you about (other) liquids that (name) may have had yesterday during the day or the night. I am interested to know whether (name) had the item even if combined with other foods.  Please include liquids consumed outside of your home.  Did (name) drink (Name of item) yesterday during the day or the night: | |  | | | | |  | | |
| --- | --- | --- | --- | --- | --- | --- | --- | --- | --- |
|  |  |  | Yes | No | DK | |  | | |
| [A] Plain water? | | Plain water | 1 | 2 | 8 | |  | | |
| [B] Juice or juice drinks? | | Juice or juice drinks | 1 | 2 | 8 | |  | | |
| [C] Soup | | Soup | 1 | 2 | 8 | |  | | |
| [CA] Tea (Tea, Cocoa, Coffee, Milo etc) | | Tea | 1 | 2 | 8 | |  | | |
| [D] Milk such as tinned, powdered, or fresh animal milk? | | Milk | 1 | 2 | 8 | |  | | |
| If yes: How many times did (name) drink milk? If 7 or more times, record '7'.  *If unknown, record ‘8’.* | | Number of times drank milk __ | | | | |  | | |
| [E] Infant formula? | | Infant formula | 1 | 2 | 8 | |  | | |
| If yes: How many times did (name) drink infant formula?  If 7 or more times, record '7'.  *If unknown, record ‘8’.* | | Number of times drank infant formula __ | | | | |  | | |
| [F] Any other liquids?    (Specify)_____________________________ | | Other liquids | 1 | 2 | 8 | |  | | |
| **BD8**. Now I would like to ask you about (other) foods that (name) may have had yesterday during the day or the night. Again, I am interested to know whether (name) had the item even if combined with other foods.  Please include foods consumed outside of your home. | | | | | | | |  | |
| Did (name) eat (Name of food) yesterday during the day or the night: | |  | Yes | No | DK | | |  |  |
| [A] Yogurt (Emasi)? | | Yogurt | 1 | 2 | 8 | | |  | |
| If yes: How many times did (name) drink or eat yogurt? If 7 or more times, record '7'. *If unknown, record ‘8’.* | | Number of times drank/ate yogurt __ | | | | | |  | |
| [B] Any cerelac, Nestum, or SIMILAR? | | Any cerelac, nestum or similar | 1 | 2 | 8 | | |  | |
| [C] Bread, rice, noodles, porridge, thin porridge (Indengane/ Incwancwa/ Inembe/ Umhidvo/ Emahewu) or other foods made from grains? | | Foods made from grains | 1 | 2 | 8 | | |  | |
| [D] Pumpkin, carrots, squash or sweet potatoes that are yellow or orange inside? | | Pumpkin, carrots, squash, etc. | 1 | 2 | 8 | | |  | |
| [E] White potatoes, white yams, manioc, cassava, or any other foods made from roots? | | White potatoes, white yams, manioc, cassava, etc. | 1 | 2 | 8 | | | |  |
| [F] Any dark green, leafy vegetables? | | Dark green, leafy vegetables | 1 | 2 | 8 | | | |  |
| [G] Ripe mangoes, papayas? | | Ripe mangoes | 1 | 2 | 8 | | | |  |
| [H] Any other fruits or vegetables? | | Other fruits or vegetables | 1 | 2 | 8 | | | |  |
| [I] Liver, kidney, heart or other organ meats? | | Liver, kidney, heart or other organ meats | 1 | 2 | 8 | | | |  |
| [J] Any meat, such as beef, pork, lamb, goat, chicken, or duck? | | Meat, such as beef, pork, lamb, goat, etc. | 1 | 2 | 8 | | | |  |
| [K] Eggs? | | Eggs | 1 | 2 | 8 | | | |  |
| [L] Fresh or dried fish or shellfish? | | Fresh or dried fish | 1 | 2 | 8 | | | |  |
| [M] Any foods made from beans, peas, lentils, or nuts? | | Foods made from beans, peas, etc. | 1 | 2 | 8 | | | |  |
| [N] Cheese or other food made from milk? | | Cheese or other food made from milk | 1 | 2 | 8 | | | |  |
| [O] Any other solid, semi-solid, or soft food that I have not mentioned?  (Specify)____________________________ | | Other solid, semi-solid, or soft food | 1 | 2 | 8 | | | |  |
| ***BD9.*** Check BD8 (Categories “A” through “O”).  **🞎** At least one “Yes” or all “DK” ⇨ Go to BD11.  **🞎** Else ⇨ Continue with BD10. | | | | | | | | | |
| **BD10**. *Probe to determine whether the child ate any solid, semi-solid or soft foods yesterday during the day or night* .  **🞎** The child did not eat or the respondent does not know ⇨ Go to Next Module.  **🞎** The child ate at least one solid, semi-solid or soft food item mentioned by the respondent ⇨ Go back to BD8 and record food eaten yesterday [A to O]. When finished, continue with BD11. | | | | | | | | | |
| **BD11**. How many times did (name) eat any solid, semi-solid or soft foods yesterday during the day or night?  If 7 or more times, record '7'. | Number of times __  DK 8 | | | | |  | | | |

| immunization IM | | | | | | | | | | |
| --- | --- | --- | --- | --- | --- | --- | --- | --- | --- | --- |
| If an immunization **(child health)** card is available, copy the dates in IM3 for each type of immunization and Vitamin A recorded on the card. IM6-IM17 will only be asked if a card is not available. | | | | | | | | | | |
| **IM1**. Do you have a card where (name)’s vaccinations are written down?  If yes: May I see it please? | | Yes, seen 1  Yes, not seen 2  No card 3 | | | | | | | | 1⇨IM3  2⇨IM6 |
| **IM2**. Did you ever have an immunization card for (name)? | | Yes 1  No 2 | | | | | | | | 1⇨IM6  2⇨IM6 |
| **IM3**.   1. Copy dates for each vaccination from the card. 2. Write ‘44’ in day column if card shows that vaccination was given but no date recorded. | | Date of Immunization | | | | | | | |  |
|  |  | Day | | Month | | Year | | | |  |
| **BCG** | **BCG** |  |  |  |  |  |  |  |  |  |
| **Polio at birth** | **OPV0** |  |  |  |  |  |  |  |  |  |
| **Polio 1** | **OPV1** |  |  |  |  |  |  |  |  |  |
| **Polio 2** | **OPV2** |  |  |  |  |  |  |  |  |  |
| **Polio 3** | **OPV3** |  |  |  |  |  |  |  |  |  |
| **Polio 4** | **OPV4** |  |  |  |  |  |  |  |  |  |
| **DPT1/hep B1/Hib1** | **DPT1/hepB1/Hib1** |  |  |  |  |  |  |  |  |  |
| **DPT2/hep B2/Hib2** | **DPT2/hepB2/Hib2** |  |  |  |  |  |  |  |  |  |
| **DPT3/hep b3/Hib3** | **DPT3/hepB3/Hib3** |  |  |  |  |  |  |  |  |  |
| **Measles** | **Measles 1** |  |  |  |  |  |  |  |  |  |
| **Measles (or MMR or MR)** | **Measles 2** |  |  |  |  |  |  |  |  |  |
| **Vitamin A** | **VitA1** |  |  |  |  |  |  |  |  |  |
| **IM4**. *Check IM3. Are all vaccines (****BCG to Measles 2****) recorded?*  **🞎** Yes ⇨ Go to IM19.  **🞎** No ⇨ Continue with IM5. | | | | | | | | | | |
| **IM5**. In addition to what is recorded on this card, did (*name*) receive any other vaccinations – including vaccinations received in campaigns or immunization days or child health days?  **🞎** Yes ⇨ Go back to IM3 and probe for these vaccinations and write ‘66’ in the corresponding day column   for each vaccine mentioned. When finished, skip to IM19.  **🞎** No/DK ⇨ Go to IM19. | | | | | | | | | | |
| **IM6**. Has (name) ever received any vaccinations to prevent him/her from getting diseases, including vaccinations received in a campaign or immunization day or child health day? | | Yes 1  No 2  DK 8 | | | | | | | | 2⇨IM19  8⇨IM19 |
| **IM7**. Has (name) ever received a BCG vaccination against tuberculosis – that is, an injection in the arm or shoulder that usually causes a scar? | | Yes 1  No 2  DK 8 | | | | | | | |  |
| **IM8**. Has (name) ever received any vaccination drops in the mouth to protect him/her from polio? | | Yes 1  No 2  DK 8 | | | | | | | | 2⇨IM11  8⇨IM11 |
| **IM9**. Was the first polio vaccine received in the first two weeks after birth? | | Yes 1  No 2 | | | | | | | |  |
| **IM10**. How many times was the polio vaccine received? | | Number of times __ | | | | | | | |  |
| **IM11**. Has (name) ever received a DPT/hep B/Hib vaccination – that is, an injection in the thigh to prevent him/her from getting tetanus, whooping cough, or diphtheria, hepatitis B or haemophilus influenzae type b disease?  Probe by indicating that DPT1/Hep B1/Hib1 vaccination is sometimes given at the same time as Polio. | | Yes 1  No 2  DK 8 | | | | | | | | 2⇨IM12A  8⇨IM12A |
| **IM12**. How many times was the DPT/hep B/Hib vaccine received? | | Number of times __ | | | | | | | |  |
| **IM16**. Has (name) ever received a Measles injection (or an MMR or MR) – that is, a shot in the arm at the age of 9 months or older - to prevent him/her from getting measles? | | Yes 1  No 2  DK 8 | | | | | | | |  |
| **IM19**. Please tell me if (name) has participated in any of the following campaigns, national immunization days and/or Vitamin A or child health days:  [A] 2013 July – integrated Measles Campaign  [B] 2014 April – PCV 13 Campaign | | Y N DK  ***Measles-2013*** 1 2 8  ***PCV13-2014*** 1 2 8 | | | | | | | |  |
| **IM20**. Issue a Questionnaire Form For Vaccination Records At Health Facility for this child. Complete the Information Panel on that Questionnaire and go to Next Module. | | | | | | | | | | |

| care of illness CA | | |
| --- | --- | --- |
| **CA1.** In the last two weeks, has (name) had diarrhoea? | Yes 1  No 2  DK 8 | 2⇨CA6A  8⇨CA6A |
| **CA2.** I would like to know how much (name) was given to drink during the diarrhoea (including breast milk).  During the time (name) had diarrhoea, was he/she given less than usual to drink, about the same amount, or more than usual?  *If less Probe*:  Was he/she given much less than usual to drink, or somewhat less? | Much less 1  Somewhat less 2  About the same 3  More 4  Nothing to drink 5  DK 8 |  |
| **CA3**. During the time (name) had diarrhoea, was he/she given less than usual to eat, about the same amount, more than usual, or nothing to eat?  *If less Probe*:  Was he/she given much less than usual to eat or somewhat less? | Much less 1  Somewhat less 2  About the same 3  More 4  Stopped food 5  Never gave food 6  DK 8 |  |
| **CA3A**. Did you seek any advice or treatment for the diarrhoea from any source? | Yes 1  No 2  DK 8 | 2⇨CA4  8⇨CA4 |
| **CA3D.** Where did you first seek advice for diarrhoea?  Probe to identify the type of source.  If unable to determine whether public or private, write the name of the place.    (Name of place) | Public sector  Government hospital 11  Government health centre 12  Government clinic/PHU 13  Rural health motivator 14  Outreach site 15  Other public (**specify**) 16  Private medical sector  Private hospital / clinic 21  Private physician 22  Private pharmacy 23  Other private medical (**specify**) 26  Other source  Relative / Friend 31  Shop 32  Traditional practitioner 33  Spiritual healer 34  Other (specify) 96 |  |
| **CA4**. During the time (name) had diarrhoea, was (name) given to drink:  [A] A fluid made from a special packet called ORS?  [B] A pre-packaged ORS fluid for diarrhoea | Y N DK  Fluid from ORS packet 1 2 8  Pre-packaged ORS fluid 1 2 8 |  |
| ***CA4A****.* Check CA4: ORS.  **🞎**  Child was given ORS (‘Yes’ circled in ‘A’ or ‘B’ in CA4) ⇨ Continue with CA4B.  **🞎** Child was not given ORS ⇨ Go to CA4C. | | |
| **CA4B**. Where did you get the ORS?  Probe to identify the type of source.  If unable to determine whether public or private, write the name of the place.    (Name of place) | Public sector  Government hospital 11  Government health centre 12  Government clinic/PHU 13  Rural health motivator 14  Outreach site 15  Other public (**specify**) 16  Private medical sector  Private hospital / clinic 21  Private physician 22  Private pharmacy 23  Other private medical (**specify**) 26  Other source  Relative / Friend 31  Shop 32  Traditional practitioner 33  Spiritual healer 34  Already had at home 40    Other (specify) 96 |  |
| **CA4C**. During the time (name) had diarrhoea, was (name) given:  [A] zinc tablets?  [B] zinc syrup? | Y N DK  Zinc tablets 1 2 8  Zinc syrup 1 2 8 |  |
| ***CA4D****.* Check CA4C: Any zinc?  **🞎**  Child given any zinc (‘Yes’ circled in ‘A’ or ‘B’ in CA4C) ⇨ Continue with CA4E.  **🞎** Child was not given any zinc ⇨ Go to CA4F. | | |
| **CA4E**. Where did you get the zinc?  Probe to identify the type of source.  If unable to determine whether public or private, write the name of the place.    (Name of place) | Public sector  Government hospital 11  Government health centre 12  Government clinic/PHU 13  Rural health motivator 14  Outreach site 15  Other public (**specify**) 16  Private medical sector  Private hospital / clinic 21  Private physician 22  Private pharmacy 23  Other private medical (**specify**) 26  Other source  Relative / Friend 31  Shop 32  Traditional practitioner 33  Spiritual healer 34  Already had at home 40    Other (specify) 96 |  |
| **CA4F**. During the time (name) had diarrhoea, was (name) given to drink home-made ORS (sugar-salt solution)? | Yes 1  No 2  DK 8 |  |
| **CA5**. Was anything (else) given to treat the diarrhoea? | Yes 1  No 2  DK 8 | 2⇨CA6A  8⇨CA6A |
| **CA6**. What (else) was given to treat the diarrhoea?  *Probe*:  Anything else?  *Record all treatments given. Write brand name(s) of all medicines mentioned.*    (Name) | Pill or Syrup  Antibiotic A  Antimotility B  Other pill or syrup (Not antibiotic, antimotility or zinc) G  Unknown pill or syrup H  Injection  Antibiotic L  Non-antibiotic M  Unknown injection N  Intravenous O  Home remedy / Herbal medicine Q  Other (*specify*) X |  |
| **CA6A.** In the last two weeks, has (name) been ill with a fever at any time? | Yes 1  No 2  DK 8 | 2⇨CA7  8⇨CA7 |
| **CA6B.**  At any time during the illness, did (name) have blood taken from his/her finger or heel for testing? | Yes 1  No 2  DK 8 |  |
| **CA7**. At any time in the last two weeks, has (name) had an illness with a cough? | Yes 1  No 2  DK 8 | 2⇨CA9A  8⇨CA9A |
| **CA8**. When (name) had an illness with a cough, did he/she breathe faster than usual with short, rapid breaths or have difficulty breathing? | Yes 1  No 2  DK 8 | 2⇨CA10  8⇨CA10 |
| **CA9**. Was the fast or difficult breathing due to a problem in the chest or a blocked or runny nose? | Problem in chest only 1  Blocked or runny nose only 2  Both 3  Other (**specify**) 6  DK 8 | 1⇨CA10  2⇨CA10  3⇨CA10  6⇨CA10  8⇨CA10 |
| ***CA9A.***  Check CA6A: Had fever?  **🞎**  Child had fever ⇨ Continue with CA10.  **🞎** *Child did not have fever ⇨ Go to CA14.* | | |
| **CA10**. Did you seek any advice or treatment for the illness from any source? | Yes 1  No 2  DK 8 | 2⇨CA12  8⇨CA12 |

| **CA11**. From where did you seek advice or treatment?  *Probe:* Anywhere else?  Circle all providers mentioned,  but do NOT prompt with any suggestions.  Probe to identify each type of source.  If unable to determine if public or private sector, write the name of the place.    (Name of place) | Public sector  Government hospital A  Government health centre B  Government clinic/PHU C  Rural health motivator D  Outreach site E  Other public (*specify*) H  Private medical sector  Private hospital / clinic I  Private physician J  Private pharmacy K  Other private medical (*specify*) O  Other source  Relative / Friend P  Shop Q  Traditional practitioner R  Spiritual healer S  Other (*specify*) X |  |
| --- | --- | --- |
| **CA12**. At any time during the illness, was (name) given any medicine for the illness? | Yes 1  No 2  DK 8 | 2⇨CA14  8⇨CA14 |
| **CA13**. What medicine was (name) given?  *Probe:*  Any other medicine?  Circle all medicines given. Write brand name(s) of all medicines mentioned.    (Name of medicines) | Anti-malarials:  SP / Fansidar A  Chloroquine B  Quinine D  Combination with Artemisinin (Coartem) . E  Mefloquine F  Other anti-malarial  (**specify**) H  Antibiotics:  Pill / Syrup I  Injection J  Other medications:  Paracetamol/ Panadol /Acetaminophen P  Aspirin Q  Ibuprofen R  Phenergan S  Cough syrup T  Other (**specify**) X  DK Z |  |
| ***CA13A****.* Check CA13: Antibiotic mentioned (codes I or J or K)?  **🞎** Yes ⇨ Continue with CA13B.  **🞎** *No* ⇨ *Go to CA13C.* | | |

| **CA13B**. Where did you get the (Antibiotic medicine from CA13)?  Probe to identify the type of source.  If unable to determine whether public or private, write the name of the place.    (Name of place) | | Home  Respondent’s home 11  Other home 12  Public sector  Government hospital 21  Government clinic / health centre/  PHU 22  Government health post 23  Government outreach site 24  Other public (**specify**) 26  Private Medical Sector  Private hospital 31  Private clinic 32  Private maternity home 33  Other private  medical (**specify**) 36  Other (specify) _____________________ 96 |  | |
| --- | --- | --- | --- | --- |
| ***CA13C.*** Check CA13: Anti-malarial mentioned (codes A - H)?  **🞎** Yes ⇨ Continue with CA13D.  **🞎** No ⇨ Go to CA14. | | | | |
| **CA13D.**  Where did you get the (Anti-Malarial medicine from CA13)?  Probe to identify the type of source.  If unable to determine whether public or private, write the name of the place.    (Name of place) | Home  Respondent’s home 11  Other home 12  Public sector  Government hospital 21  Government clinic / health centre/  PHU 22  Government health post 23  Government outreach site 24  Other public (**specify**) 26  Private Medical Sector  Private hospital 31  Private clinic 32  Private maternity home 33  Other private  medical (**specify**) 36  Other (specify) _______________________ 96 | | |  |
| **CA13E.** How long after the fever started did (name) first take (name of anti-malarial from CA13)?  *If multiple anti-malarials mentioned in CA13, name all anti-malarial medicines mentioned.* | Same day 0  Next day 1  2 days after the fever 2  3 days after the fever 3  4 or more days after the fever 4  DK 8 | | |  |

| ***CA14****.* Check AG2: Age of child.  **🞎** Child age 0, 1 or 2 ⇨ Continue with CA15.  **🞎** Child age 3 or 4 ⇨ Go to UF13. | | |
| --- | --- | --- |
| **CA15**. The last time *(*name*)* passed stools, what was done to dispose of the stools? | Child used toilet / latrine 01  Put / Rinsed into toilet or latrine 02  Put / Rinsed into drain or ditch 03  Thrown into garbage (solid waste) 04  Buried 05  Left in the open 06  Other (**specify**) 96  DK 98 |  |

| **UF13**. *Record the time.* | Hour and minutes __ __ : __ __ |  |
| --- | --- | --- |

| ***UF14****.* Check List of Household Members, columns HL7B and HL15.  Is the respondent the mother or caretaker of another child age 0-4 living in this household?  **🞎** Yes ⇨ Indicate to the respondent that you will need to measure the weight and height of the child  later. Go to the next QUESTIONNAIRE FOR CHILDREN UNDER FIVE to be  administered to the same respondent.  **🞎** No ⇨ End the interview with this respondent by thanking her/him for her/his cooperation and  tell her/him that you will need to measure the weight and height of the child before you  leave the household.  Check to see if there are other woman’s, man’s or under-5 questionnaires to be  administered in this household. |
| --- |

| anthropometry AN | | |  |
| --- | --- | --- | --- |
| After questionnaires for all children are complete, the measurer weighs and measures each child.  Record weight and length/height below, taking care to record the measurements on the correct questionnaire for each child. Check the child’s name and line number in the List of Household Members before recording measurements. | | |  |
| **AN1**. *Measurer’s name and number:* | Name ___ ___ |  |  |
| **AN2**. *Result of height / length and weight measurement:* | Either or both measured 1  Child not present 2  Child or mother/caretaker refused 3  Other (**specify**) 6 | 2⇨AN6  3⇨AN6  6⇨AN6 |  |
| **AN3**. *Child’s weight:* | Kilograms (kg) __ __ . __  Weight not measured 99.9 |  |  |
| **AN3A**. *Was the child undressed to the minimum?*  **🞎** Yes.  **🞎** No, the child could not be undressed to the minimum. | | |  |
| **AN3B**. *Check age of child in AG2:*  **🞎** Child under 2 years old ⇨ Measure length (lying down).  **🞎** Child age 2 or more years ⇨ Measure height (standing up). | | |  |
| **AN4**. *Child’s length or height:* | Length / Height (cm) __ __ __ . __  Length / Height not measured 999.9 | ⇨ AN6 |  |
| **AN4A**. *How was the child actually measured? Lying down or standing up?* | Lying down 1  Standing up 2 |  |  |
|  | | |  |
| ***AN6****.* Is there another child in the household who is eligible for measurement?  **🞎** Yes ⇨ Record measurements for next child.  **🞎** No ⇨ Check if there are any other individual questionnaires to be completed in the household. | | |  |
| **Interviewer’s Observations** | | | |
|  | | | |

| **Field Editor’s Observations** |
| --- |
|  |

| **Supervisor’s Observations** |
| --- |
|  |

| **Measurer’s Observations** |
| --- |
|  |
